# Lactic Acid Bacteria Isolated from Fermented Doughs in Spain Produce Dextrans and Riboflavin

**DOI:** 10.3390/foods10092004

**Published:** 2021-08-26

**Authors:** María Goretti Llamas-Arriba, Annel M. Hernández-Alcántara, Mari Luz Mohedano, Rosana Chiva, Lorena Celador-Lera, Encarnación Velázquez, Alicia Prieto, María Teresa Dueñas, Mercedes Tamame, Paloma López

**Affiliations:** 1 Departamento de Química Aplicada, Facultad de Químicas, Universidad del País Vasco (UPV/EHU), 20018 San Sebastián, Spain; gorettillamas@gmail.com (M.G.L.-A.); mariateresa.duenas@ehu.eus (M.T.D.); 2 Departamento de Biotecnología Microbiana y de Plantas, Centro de Investigaciones Biológicas Margarita Salas (CIB, CSIC), 28040 Madrid, Spain; annel@cib.csic.es (A.M.H.-A.); mmoheda@cib.csic.es (M.L.M.); aliprieto@cib.csic.es (A.P.); 3 Instituto de Biología Funcional y Genómica, (IBFG, CSIC), Universidad de Salamanca, 37007 Salamanca, Spain; rosanachiva@usal.es (R.C.); tamame@usal.es (M.T.); 4 Departamento de Microbiología y Genética, Universidad de Salamanca, 37007 Salamanca, Spain; lorenacelador@usal.es (L.C.-L.); evp@usal.es (E.V.); 5 Unidad Asociada USAL-IRNASA (CSIC), 37007 Salamanca, Spain

**Keywords:** lactic acid bacteria, dextrans, riboflavin, sourdoughs

## Abstract

Many lactic acid bacteria (LAB) produce metabolites with applications in the food industry, such as dextran-type exopolysaccharides (EPS) and riboflavin (vitamin B_2_). Here, 72 bacteria were isolated from sourdoughs made by Spanish bread-makers. In the presence of sucrose, colonies of 22 isolates showed a ropy phenotype, and NMR analysis of their EPS supported that 21 of them were dextran producers. These isolates were identified by their random amplified polymorphic DNA (RAPD) patterns and their *rrs* and *pheS* gene sequences as LAB belonging to four species (*Weissella cibaria*, *Leuconostoc citreum*, *Leuconostoc falkenbergense* and *Leuconostoc mesenteroides*). Six selected strains from the *Leuconostoc* (3) and *Weissella* (3) genera grew in the absence of riboflavin and synthesized vitamin B_2_. The EPS produced by these strains were characterized as dextrans by physicochemical analysis, and the *L. citreum* polymer showed an unusually high degree of branching. Quantification of the riboflavin and the EPS productions showed that the *W. cibaria* strains produce the highest levels (585–685 μg/and 6.5–7.4 g/L, respectively). Therefore, these new LAB strains would be good candidates for the development of fermented foods bio-fortified with both dextrans and riboflavin. Moreover, this is the first report of riboflavin and dextran production by *L. falkenbergense*.

## 1. Introduction

Lactic acid bacteria (LAB) occur naturally in fermented foods, such as “mother doughs” (also named Type I sourdoughs) or bakery doughs from bakers’ stores. The microorganisms and their metabolites isolated from these fermented matrices are classified as qualified presumption of safety (QPS), generally regarded as safe (GRAS), and considered as non-toxic and food-grade microorganisms [1].

Spontaneous sourdoughs inoculated into only flour and water are fermented by plant and/or cereal matrix-associated microbiota. The most prevalent initial bacterial species belong to the family Enterobacteriaceae, which are later replaced by more acid-tolerant *Weissella* and *Leuconostoc* species, until a number of different lactobacilli prevail [2,3].

The metabolic transformations carried out by LAB are potentially an important biotechnological process for the functionalization and/or nutritional fortification of breads. Thus, great attention is given to the discovery and characterization of new LAB strains that are able to biosynthesize postbiotics [4] such as exopolysaccharides (EPS) to exploit their functional properties in foods, especially in bread [5,6,7,8,9]. Many LAB strains of different species produce EPS, especially dextrans with a linear backbone mainly composed of D-glucopyranosyl residues with α-(1→6) linkages and varying percentages of α-(1→4), α-(1→3), or α-(1→2) branches [10], which can act as immunomodulators [11] or antivirals [12]. In addition, the EPS produced in situ by some LAB species are of interest because of their contribution to the rheology of the doughs and to the texture of the products, especially in gluten-free breads [7,8,13,14].

Apart from dextrans, LAB can also be a valuable source for vitamin biofortification of sourdoughs. Some of these vitamins are found as precursors of intracellular co-enzymes (e.g., riboflavin (vitamin B_2_) and precursors of flavin mononucleotide (FMN) and flavin adenine dinucleotide (FAD)), which are necessary for the regulation of biochemical reactions (oxidation–reduction) in the cell [15]. Currently, thirteen vitamins have been recognized as being essential to human health: fat-soluble vitamins (A, D, E, and K) and water-soluble (vitamin C and B-group vitamins). As humans are not capable of synthesizing most vitamins, they have to be obtained exogenously [16]. B-group vitamins (thiamine, riboflavin, niacin, pyridoxine, pantothenic acid, biotin, folate, and cobalamin) differ chemically but act in synergy to maintain the body’s homeostasis [15].

Nowadays, B vitamin deficiencies occur as a result of non-balanced and unvaried diets, mainly in developing countries, whose populations’ staple foods are cereals and pseudo-cereals. In addition, processing and cooking of the cereals increases the loss of a large portion of B-group vitamins, such as vitamin B_2_, folates, or thiamine, [17,18]. Therefore, many countries have adopted mandatory fortification programs with specific vitamins and minerals. Due to the above, the use of vitamin-producing microorganisms could be considered a cost-effective method to produce fermented foods with high concentrations of naturally synthesized vitamins and thereby avoid the undesirable side effects associated with chemically synthesized vitamins that are used in the fortification of food [16,19].

Some LAB species such as *Lactococcus lactis*, *Lactobacillus gasseri*, and *Lactobacillus reuteri*, as well as *Bifidobacterium adolescentis* are able to produce B vitamins in high quantities in fermented foods [20,21], and various other strains isolated from a wide variety of niches were successfully employed to improve the B-group vitamins content of foods [22,23]. Recently, riboflavin fortification using selected riboflavin-producing LAB strains in food matrices such as milk, soymilk, whey, kefir-like cereal-based beverages, and pseudo-cereals was reported [18,24,25,26,27]. Additionally, bread and pasta are emerging as functional foods, because their characteristics allow them to be enriched in vitamins [28]. Therefore, the use of riboflavin-producing LAB to bio-enrich foods represents a more natural and consumer-friendly alternative to the use of chemically synthesized pseudo-vitamins [16,29].

In this context, this work is focused on the search, isolation from different sourdoughs, and characterization of dextran and riboflavin-producing LAB, with the future aim of formulating defined microbial consortia to produce functional bio-fortified breads.

## 2. Materials and Methods

### 2.1. Bacterial Isolation

Seventy-two LAB isolates were recovered from samples provided by various bread-makers. Sixty-four strains were recovered from mother doughs (MD) obtained by spontaneous fermentations and back-slopping procedures: 45 from MD22 made of rye flour and 19 from MD21 made of wheat flour. Moreover, 8 were recovered from a bakery dough (BD16). The LAB strains recovered from MD21 and BD16 [30] belonged to the shared “PANBAL” collection (IBFG-CSIC Institute and the University of Salamanca, Salamanca, Spain) and those from MD22 belonged to the IBFG-CSIC collection. Of these, 19 from MD22, 1 from MD21, and 2 from BD16 (Table S1) showed a ropy phenotype in MRS medium (Man, Rogosa and Sharpe medium, Sigma Co, Darmstadt, Germany) supplemented with 5% sucrose, and they were studied in this work.

For LAB isolation, samples of 10 g of each sourdough were homogenized in 90 mL of sterile peptone water (1 g/L peptone, 8.5 g/L NaCl) in 250 mL flasks and incubated at 28 °C for 1 h with shaking (200 rpm). Then, 5 mL samples were centrifuged (3000× *g*), the supernatants were diluted 10-fold, and 0.1 mL aliquots were spread on MRS-agar plates containing 2% glucose (MRSG-agar). The plates were incubated at 28 °C for 4 days. Individual colonies were transferred to MRS-agar plates containing 5% sucrose to detect the ropy phenotypes, which were classified as strong, normal, and light on the basis of their mucous appearance on this medium (Table S1 and Figure S1).

### 2.2. DNA Extraction and Random Amplified Polymorphic DNA (RAPD) Pattern Analysis

For RAPD fingerprinting, the total DNA was extracted from bacterial cells grown on MRSG-agar plates for 48 h at 28 °C, as described by Rivas et al. [31]. Using the total DNA as a template, the RAPD patterns were obtained by the Polymerase Chain Reaction (PCR) using the M13 primer (5′-GAGGGTGGCGGTTCT-3′) and Dream Taq-polymerase (Thermo Fisher, Madrid, Spain) [31]. PCR conditions were as follows: preheating at 95 °C for 10 min, 39 cycles of denaturing at 94 °C for 1 min, annealing at 45 °C for 1 min, extension at 72 °C for 2 min, and a final extension step at 72 °C for 7 min. The PCR products were conserved at 5 °C, and 10 µL of each sample were electrophoresed on 1.5% (*w*/*v*) agarose gel in TBE buffer (100 mM Tris, 83 mM boric acid, 1 mM ethylenediaminetetraacetic acid EDTA, pH 8.5) at 6 V/cm, stained in a solution containing 0.5 μg/mL ethidium bromide, and photographed using a Gel Doc XR (Bio-Rad Laboratories, S.A., Alcobendas, Spain). (GeneRuler 1 kbp Plus DNA Ladder (Thermo Fisher Scientific Madrid, Spain) was used as a size marker.

### 2.3. Characterization of rrs and pheS Genes

**Figure 1:**
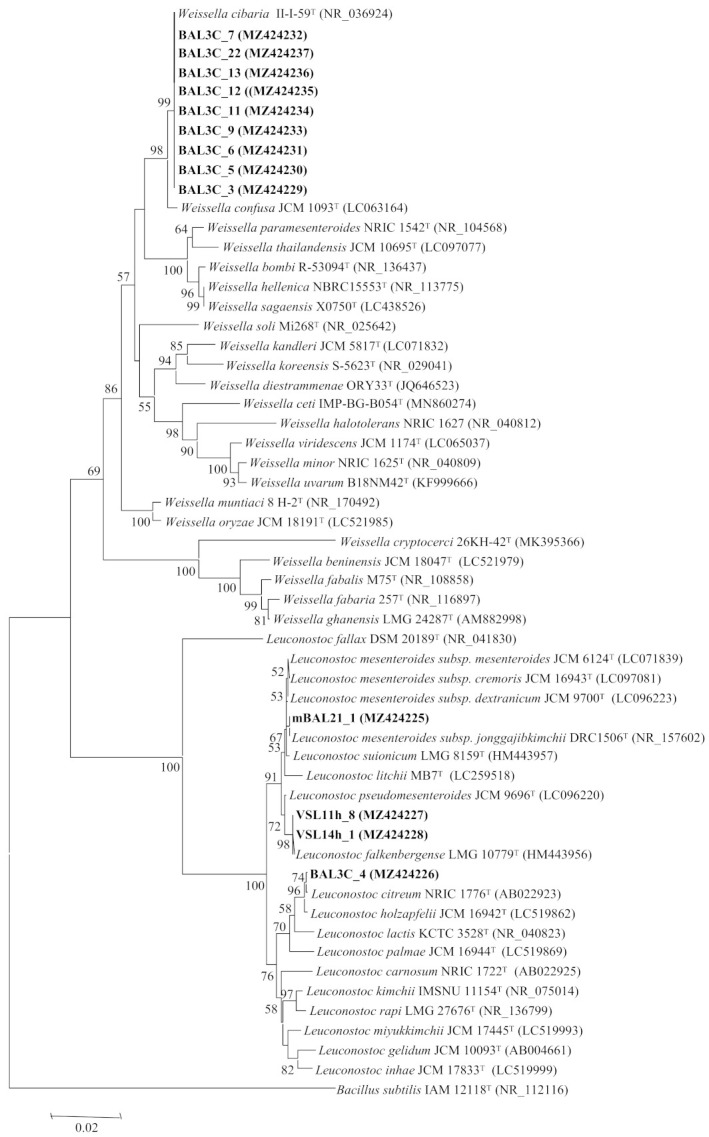
Neighbour joining phylogenetic rooted tree based on the partial sequences (1400 nt) of the 16S rRNA coding gene, showing the taxonomic location of the analysed strains. Bootstrap values calculated for 1000 replications are indicated. Bar, 1 nt substitution per 100 nt. Accession numbers from GenBank are given in brackets.

**Figure 2:**
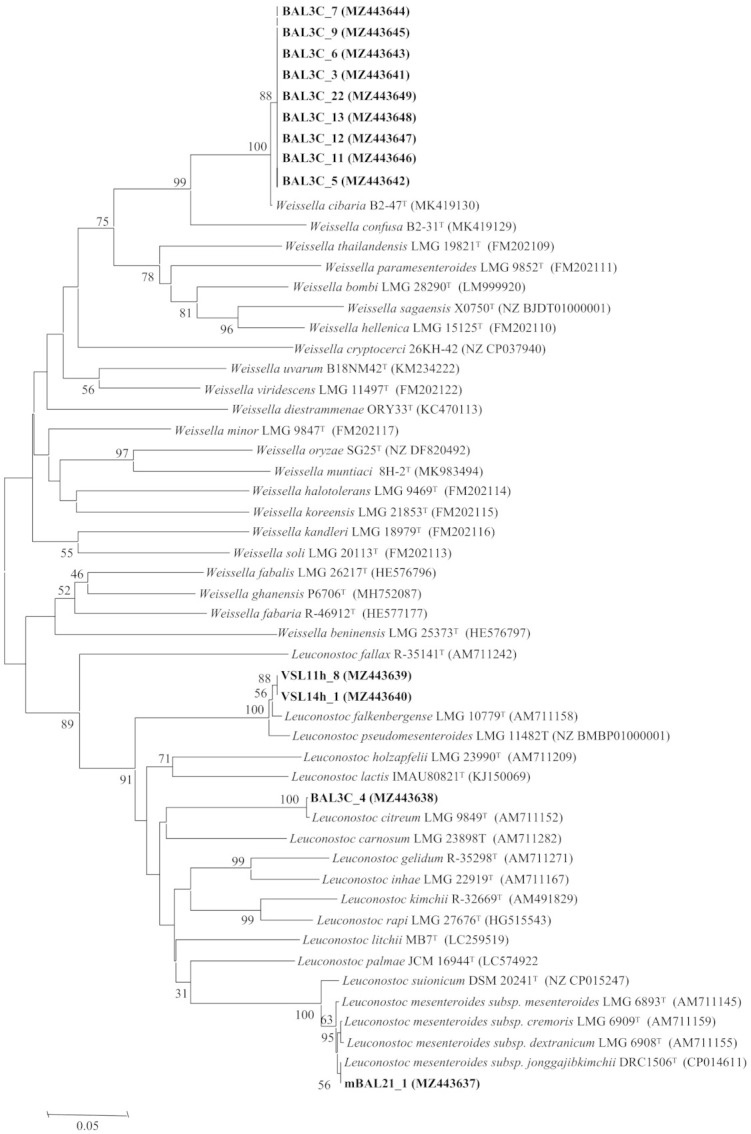
Neighbour joining phylogenetic unrooted tree based on the *pheS* gene partial sequences (340 nt) showing the taxonomic location of the analysed strains. Bootstrap values calculated for 1000 replications are indicated. Bar, 5 nt substitution per 100 nt. Accession numbers from GenBank are given in brackets.

The amplification and sequencing of the *rrs* gene, encoding the 16S rRNA, was carried out as indicated by Carro et al. [32] using the primers SF1 (5′-AGAGTTTGATCMTGGCTCAG-3′) and 1522R (5′-AAGGAGGTGATCCANCC-3′) for amplification, as well as the primer SR4 (5′-GGGTTGCGCTCGTTG-3′) for sequencing. The amplification and sequencing of the *pheS* gene, encoding the α-subunit of phenylalanyl-tRNA synthetase, was carried out as indicated by Naser et al. [33] using the primers *pheS*-21-F (5′-CAYCCNGCHCGYGAYATGC-3′) and *pheS*-23-R (5′-GGRTGRACCATVCCNGCHCC-3′). The sequencing of these genes was performed by the Sequencing DNA Service at the NUCLEUS platform of Salamanca University (Salamanca, Spain). The obtained sequences were deposited in GenBank (see accession numbers in Figure 1 and Figure 2) and compared with those from the type strains held in GenBank using the BLASTN program [34]. Sequences were aligned using the ClustalX software [35]. The distances were calculated according to Kimura’s two-parameter model [36]. Phylogenetic trees were inferred using the neighbour joining (NJ) analysis [37]. Molecular Evolutionary Genetic Analysis version MEGA7 software [38] was used for all these analyses.

### 2.4. Detection of EPS Production on Solid Media and by Transmission Electron Microscopy (TEM)

LAB strains were grown in liquid MRSG medium to the middle of the exponential phase. Then, 100 µL aliquots of 10^−6^ dilutions of the cultures were spread on either MRSS- or MRSG-agar plates and incubated at 30 °C for 72 h to visualize the colonies’ phenotypic evolution.

For TEM analysis, bacteria grown on MRSG or MRS supplemented with 2% sucrose (MRSS) solid media were used. After 48 h, one colony of each LAB was dispensed, individually, with a micropipette tip into an Eppendorf tube containing ultrapure water (150 µL). The colonies were carefully resuspended with the tip without vortexing, and 10 µL of each suspension were deposited onto carbon film copper grids (Formvar/carbon 400 mesh), which were pre-treated as previously described [39]. The grids were stained for 10 s with an aqueous uranyl acetate solution (0.1% *w*/*v*) and rinsed with water. Finally, the preparations were examined in a JEOL JEM-1230 electron microscope (JEOL Ltd., Tokio, Japan) operating at an acceleration voltage of 100 kV.

### 2.5. Production, Purification, and Quantification of Dextran

For the initial NMR characterization of the 21 LAB EPS, bacteria were grown first in MRSS liquid medium at 28 °C for 24 h, and the cultures were used to inoculate 5 mL of a semi-defined medium (SMD, [40]) containing 2% sucrose (SMDS) to give an initial optical density at 600 nm (OD_600nm_) of 0.2, and these were grown for 48 h at 28 °C.

For further physicochemical characterization of the EPS from the 6 selected LAB strains, the bacteria were grown in MRSS liquid medium for 20 h, and the cultures were used to inoculate RAMS (20 mL) to give an initial OD_600nm_ of 0.1 and grown from 24 h at 30 °C.

After growing the LAB as described above, bacteria were sedimented by centrifugation at 7300× *g* for 1 h. Then, the EPS were isolated as described previously [41]. Briefly, the culture supernatants were mixed with 95% ethanol (1:1 *v*/*v*) and maintained at 4 °C for 20 h. The precipitated EPS were sedimented by centrifugation at 7300× *g* for 1 h. After supernatant removal, the precipitates were dried; then, they were resuspended in and dialyzed against ultrapure water for 24 h using a membrane with a 12–14 kDa cut-off. Then, the EPS preparations were frozen at −80 °C and lyophilized for 72 h. The dry biopolymers were kept at room temperature prior to further analyses. Suspensions of EPS preparations in ultrapure water at 1 mg/mL were used to analyse the purity of the EPS by determining the concentration of contaminating DNA, RNA, and proteins by fluorescence measurement using specific kits and the Qubit 2.0 fluorometric detection methods (Thermo Fisher Scientific, Waltham, MA, USA). Quantification of the EPS was performed from culture supernatants and from suspensions of the purified EPS by measurement of the total sugar content by the phenol–sulfuric method [42].

### 2.6. Characterization of the EPS

Purified EPS preparations from the 6 selected LAB were characterized by (i) identification of their neutral sugars, (ii) methylation analysis, and (iii) interpretation of the Fourier transform infrared (FT-IR) spectra, as previously described [43]. Furthermore, the EPS from the 22 initially selected LAB strains were analysed by one-dimensional nuclear magnetic resonance (1D, ^1^H-NMR). Samples were weighed (ca. 1 mg) and dissolved 1:1 (*w*/*v*) in 99% D_2_O, and their spectra were recorded at 298 K using a Bruker Avance NEO spectrometer (Bruker, Bremen, Germany) operating at 500.13 MHz (^1^H). Chemical shifts are given in ppm using the deuterated water signal (4.70 ppm) as reference. Spectra were acquired with pre-saturation of the residual deuterium hydrogen oxide (HDO) signal.

### 2.7. Analysis of Bacterial Growth and Riboflavin Production and Quantification

LAB strains were grown overnight in MRSG and sedimented by centrifugation at 9300× *g* for 20 min at room temperature. Afterwards, they were resuspended in either the riboflavin assay medium (RAM, Difco) supplemented with 2% sucrose (RAMS) or the RMAS supplemented with 2% riboflavin (RAMSR) to give an OD_600nm_ of 0.1. Then, aliquots of 200 µL of each culture were analysed in duplicate in sterile 96-well optical white w/lid cell culture polystyrene plates (Thermo Fisher Scientific). The growth at 30 °C and the riboflavin fluorescence of the bacterial cultures in real time (measurements every 30 min) was performed using a Varioskan Flask System (Thermo Fisher Scientific), as previously described by Mohedano et al. [44]. Growth was monitored at OD_600nm_, and the riboflavin fluorescence was measured upon excitation at a wavelength of 440 nm and detection of emission at a wavelength of 520 nm.

Quantification of the riboflavin concentration was performed as previously described [30] by using a calibration curve constructed to correlate the fluorescence emitted by solutions prepared in RAM with increasing concentrations of riboflavin at 520 nm. The growth rate (µ) of the LAB in liquid media was determined as described by Widdle [45].

### 2.8. Statistical Analysis

Riboflavin and EPS production (after checking that ANOVA assumptions were met, i.e., normality of the residuals and heteroscedasticity, differences between groups) were tested with a one-way analysis of variance. A *p*-value of ≤0.05 was considered significant. Mean pairwise comparisons were computed with a Tukey’s test (α = 0.05), and the results are shown with letters; means with the same letter are not significantly different. All analyses were performed with the R software version 3.6.3 (R Fundation for Statitical Computing, Vienna, Austria) [46].

## 3. Results and Discussion

### 3.1. Isolated Strains

With the aim to detect and characterize EPS and riboflavin-producing LAB strains, 72 isolates were recovered from samples provided by different bread-makers. The ropy visual and tactile appearance of the bacterial grown on solid medium is the most commonly used method to select strains producing different types of EPS [47]. Therefore, these strains were screened for EPS production by cultivation on a modified MRS-agar medium in order to detect and to evaluate their potential “ropy” phenotype or mucoid visual appearance. Among them, only 22 LAB (Table S1) showed different degrees of “ropiness” (Figure S1); therefore, these strains were selected for further taxonomical classification by DNA typing.

### 3.2. RAPD Fingerprinting

RAPD fingerprinting has been widely used for LAB characterization, and in the past year, this methodology has been used in several works to analyse their diversity in different sourdoughs [9,48,49,50,51]. Therefore, in this work, we used this methodology to assess the genetic diversity of the strains showing a ropy phenotype, and the results obtained indicated that the tested strains presented 13 different RAPD pattern types (Figure S2 and Table 1). Therefore, a strain representative of each one of these types of RAPD patterns was selected for further identification.

### 3.3. Analysis of the rrs and pheS Gene Sequences

The classification of LAB is based on their *rrs* genes, which encode their 16S rRNAs [52] and therefore, the DNA sequence of the genes from the 13 selected strains was determined. The obtained results revealed that they belong to two LAB genera, *Leuconostoc* and *Weissella*, which are commonly found in bread sourdoughs [2,3]. The strains VSL11h-8 and VSL14h-1 (RAPD types A and B, respectively) possessed the same gene sequence, which was identical to that of the type-strain *Leuconostoc falkenbergense* LMG 10779^T^. In addition, the gene sequences of mBAL21_1 and BAL3C-4 (RAPD types C and D, respectively) showed 99.9% similarity with those of *Leuconostoc mesenteroides* JCM 6124^T^ and *L. citreum* JCM 9698^T^, respectively. The remaining strains carried *rrs* with 99.9% similarity to that of the type-strain *Weissella cibaria* JCM 12495^T^ (Table 1).

Although all these similarity values were equal to or near 100%, it was necessary to confirm the identification through the analysis of other phylogenetic markers with higher interspecific variability, because the genera *Leuconostoc* and *Weissella* encompass several closely related species, some of them containing different subspecies, whose clear differentiation is not possible using only the 16S rRNA coding gene analysis (Figure 1). For example, in the genus *Leuconostoc*, the type strains of several species showed 99.5% similarity values in this gene, as occurs also between *L. falkenbergense* and *L. pseudomesenteroides*, between *L. suionicum* and the four subspecies of *L. mesenteroides*, and between *L. citreum* and *L. holzapfelii* (Figure 1). The genus *Weissella* also includes several species with similarities higher than 99.5%, as occurs between *W. cibaria* and *W. confusa* type strains (Figure 1).

To confirm the identification of the strains from this study, we analysed the *pheS* gene, which encodes the α-subunit of phenylalanyl-tRNA synthetase, which has been used to differentiate LAB species, particularly in the case of the two genera found in this study, *Leuconostoc* and *Weissella* [53,54] and among the subspecies of the same species, such as those from *L. mesenteroides* [55]. The results are depicted in Table 1 and Figure 2, and they confirmed the identification of BAL strains (from bakery Val de San Lorenzo) VSL11-h8 and VSL14h-1 as *L. falkenbergense*. VSL11h-8 and VSL14h-1 as *L. falkenbergense* with 99.2% similarity and that of BAL3C-4 as *L. citreum* with 100% similarity. In the case of mBAL21_1, the *pheS* gene analysis indicated its affiliation to *L. mesenteroides* subsp. *jonggajibkimchii* with 100% similarity in this gene. Concerning the strains belonging to the genus *Weissella*, the *pheS* gene analysis confirmed that they belong to the species *W. cibaria* with 98.9% similarity (Table 1, Figure 2). The species *L. citreum* [56,57,58,59,60,61,62] and *W. cibaria* [56,63,64] are commonly found in sourdoughs, but as far as we know, this is the first report describing the presence in bread sourdoughs of *L. mesenteroides* subsp. *jonggajibkimchii* and *L. falkenbergense*, which is a recently described species whose type strains were previously included in the species *L. mesenteroides* [65].

### 3.4. Detection of EPS Production by LAB Strains

Dextran is synthesized by dextran sucrases using sucrose, but not glucose, as substrate. Therefore, with the aim of detecting dextran-producing strains, the EPS production of the LAB isolates was investigated. The *L. mesenteroides* subsp. *jonggajibkimchii* mBAL21_1 was not included due to its low EPS production (Figure S1).

Thus, the 21 LAB strains were grown in SDM medium containing 2% sucrose and lacking polysaccharides. Then, the EPS present in the supernatants were quantified and, after purification, subjected to ^1^H-NMR analysis (Figure S3). The spectra revealed that, indeed, the 21 strains produced dextran, as all of them displayed two characteristic peaks: a signal at 4.90 ppm from the α-(1→6) glucopyranose backbone and a minor one at 5.3 ppm from the α-(1→3) side chains. Consequently, only six strains were selected for further analyses on the basis of the species group to which they belonged (Table 1) as well as different RAPD patterns (Figure S2). Thus, the two *L. falkenbergense* (VSL11h-8 and VSL14h-1; RAPD A and RAPD B types) and *L. citreum* BAL3C-4 (RAPD D) were selected. In addition, among the nine RAPD patterns types detected for the *W. cibaria* strains, only three BAL were selected (BAL3C-5, BAL3C-7, and BAL3C-22; RAPD F, RAPD H, and RAPD M), which produce in SDM medium (Figure S3) different levels of EPS (0.8, 0.5, and 0.3 g/L, respectively) and belong to a different RAPD group.

To analyse phenotypically the EPS production, the six selected LAB strains were grown on plates containing either MRSS-agar or MRSG-agar supplemented respectively with sucrose or glucose as a carbon source. The six bacteria generated mucous colonies only in the presence of the disaccharide, as previously observed for other dextran-producing *Leuconostoc* and *Weissella* strains [11,41] and Figure S4). The production of the EPS by the colonies increased during the 72 h of incubation at 30 °C, and in the case of *W. cibaria* strains, a mucous layer was formed on the top of the plate (Figure S4). By contrast, when glucose was present, smaller non-mucous colonies were observed even after the whole incubation period (Figure S4).

**Figure 3:**
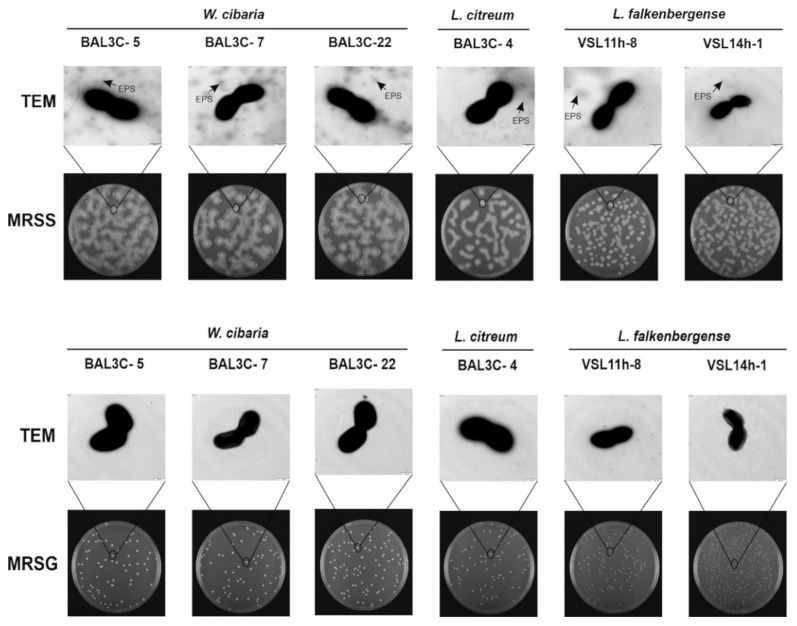
Detection of exopolysaccharide EPS production by the indicated lactic acid bacteria at the macroscopic and microscopic levels. Appearance of bacterial colonies after growth on MRS-agar supplemented with either 2% sucrose (MRSS) or 2% glucose (MRSG) for 48 h at 30 °C as well as colony analysis by transmission electron microscopy (TEM) are depicted.

**Figure 4:**
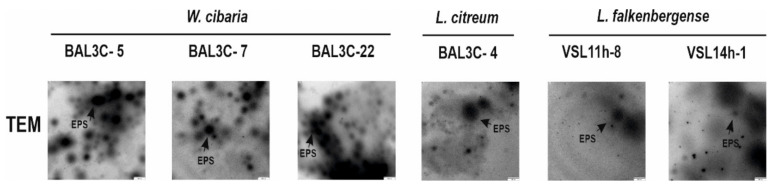
Detection by TEM of the EPS production by LAB. Pictures of the EPS present in samples from LAB colonies after 48 h of growth in MRSS.

Furthermore, after 48 h of incubation, colonies were observed by TEM, and EPS was detected surrounding the bacterial cells (Figure 3), as we have previously described for other *W. cibaria* and *Leuconostoc* strains [11,41,66]. In addition, unattached EPS was also detected (Figure 4).

As expected, EPS was not found in the TEM images for any of the tested strains grown in MRSG (Figure 3). Consequently, these results supported that the EPS produced by the six selected LAB strains was dextran-type.

### 3.5. Influence of Carbon Source on LAB Growth

**Figure 5:**
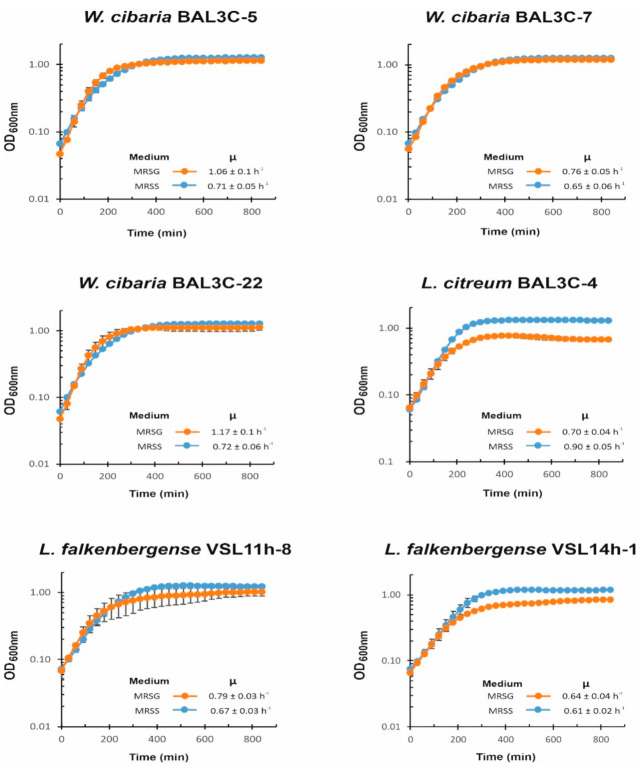
Influence of the presence of sucrose on lactic acid bacteria growth. A comparative analysis in real time of culture growth in MRS medium supplemented with either 2% sucrose (MRSS) or 2% glucose (MRSG), as well as the inferred growth rate during exponential phase, are depicted.

Dextran sucrases hydrolyse sucrose, generating dextran with a concomitant release of fructose, which can be used by the bacteria as a carbon source, and our previous results showed a preference of sucrose vs. glucose as a carbon source for the growth of *W. cibaria* AV2ou isolated from avocado [66]. Therefore, to evaluate the influence of these two carbohydrates on the growth of LAB isolated from doughs, first, growth in MRSS and MRSG was monitored overnight at 30 °C (Figure 5).

For the three *W. cibaria* strains, the presence of sucrose did not have a positive influence on the bacterial growth rate during the exponential phase, and the LAB reached a similar final OD_600nm_ at the stationary phase in both the MRSS or MRSG medium. However, for *L. citreum* BAL3C-4, the presence of sucrose was advantageous, reaching at the stationary phase a biomass estimated by the OD_600nm_ values of ca. 0.5 higher upon growth in MRSS vs. MRSG, together with a higher growth rate during the exponential phase in the presence of the disaccharide (0.9 vs. 0.7). In the case of the two *L. falkenbergense* strains, the presence of sucrose allowed them to reach higher values of OD_600nm_ prior to entry into the stationary phase than in the presence of glucose, but the growth rate was either lower in MRSS than in MRSG for VSL11h-8 (0.67 vs. 0.79) or almost not affected by the carbon source for VSL14h-1 (0.61 vs. 0.64). Therefore, the utilization of sucrose seems to allow *L. citreum* and *L. falkenbergense* strains to reach a higher biomass under these growth conditions.

### 3.6. Analysis of Riboflavin Production by LAB during Growth

**Figure 6:**
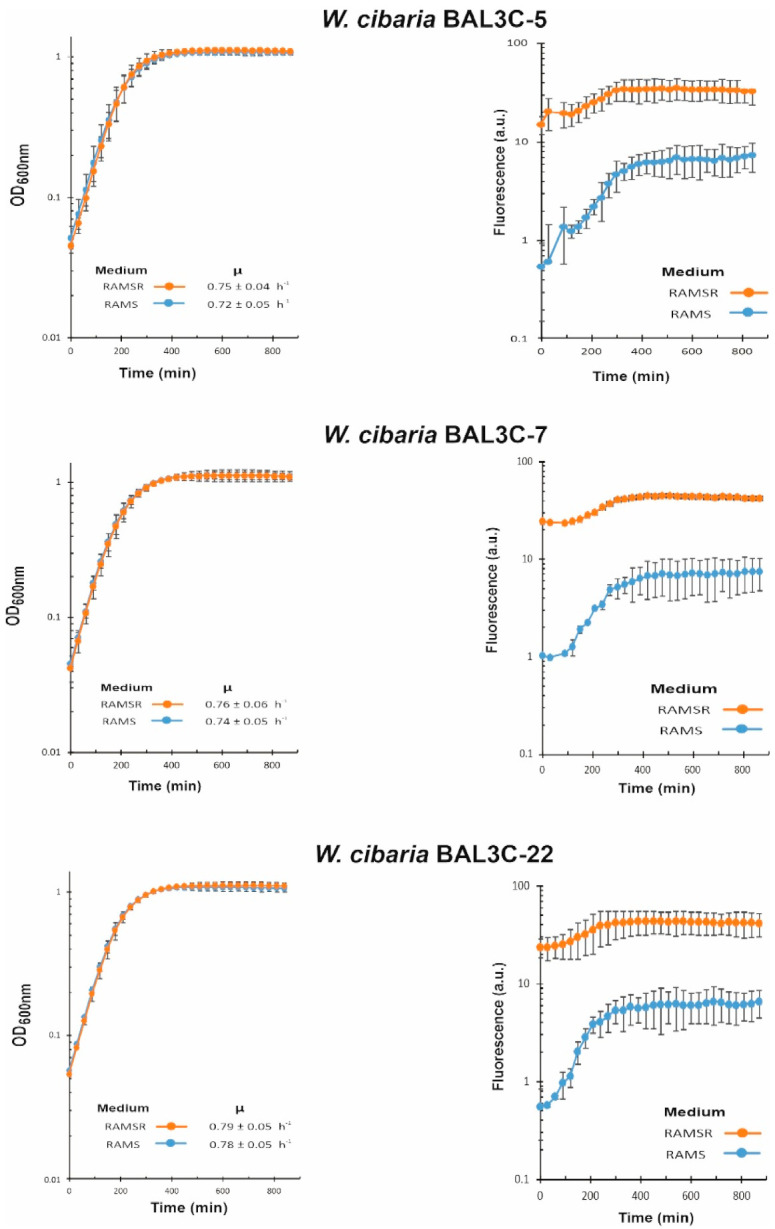
Detection of riboflavin production by the *W. cibaria* strains. A comparative analysis in real time of lactic acid bacteria cultures grown in RAM medium supplemented with either 2% sucrose (RAMS) or 2% sucrose plus 2% riboflavin (RAMSR) is depicted. Left panels show the growth and growth rate of the cultures during the exponential phase. Right panels show the detection of riboflavin fluorescence.

**Figure 7:**
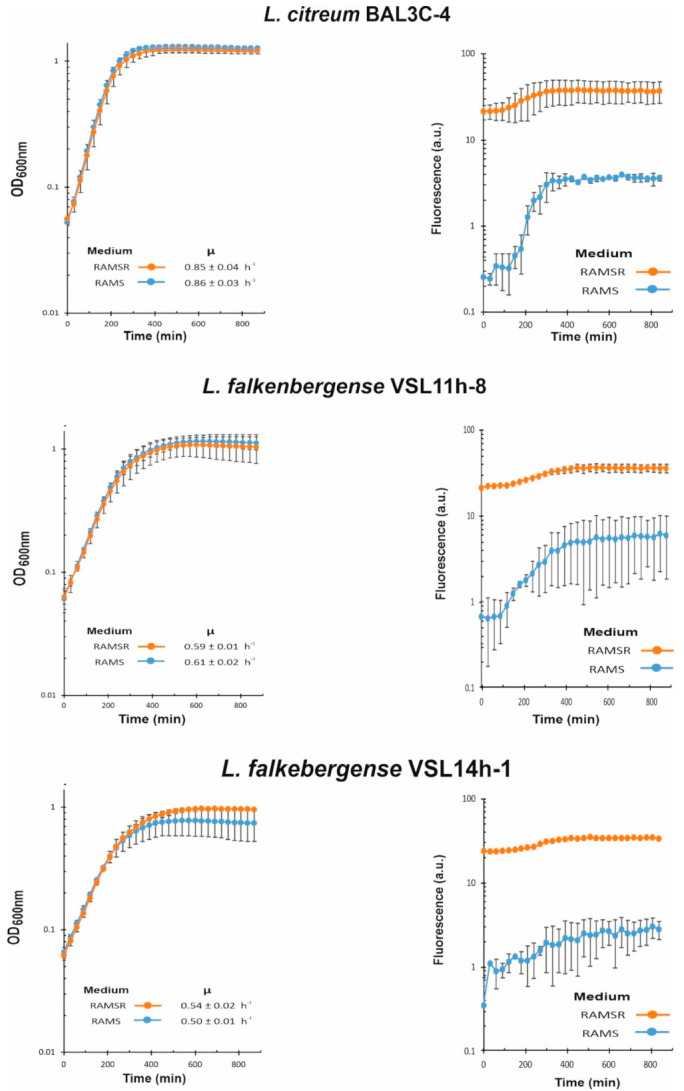
Detection of riboflavin production by *Leuconostoc citreum* BAL3C-4 and the *L. falkerbengense* strains. A comparative analysis in real time of lactic acid bacteria cultures grown in in RAM medium supplemented with either 2% sucrose (RAMS) or 2% sucrose plus 2% riboflavin (RAMSR) is depicted as in Figure 6.

We have recently developed a method to detect and quantify the production of riboflavin in real time during growth by measuring the fluorescence of the flavin [44]. Moreover, the RAM medium without supplementation with riboflavin only supports the growth of riboflavin-producing strains. Thus, with the aim to test if the six selected LAB strains were riboflavin producers and to assess levels of EPS production in the presence of sucrose, their growth in RAMS and in RAMSR (supplemented with riboflavin) was monitored as well as the fluorescence emitted in both media (Figure 6 and Figure 7).

EPS and riboflavin levels present in the supernatants of the LAB cultures after growth for 22 h at 30 °C in RAMS were also quantified (Table 2). The six LAB strains were able to grow in the absence of riboflavin, and production of the vitamin was detected in RAMS and RAMSR (Figure 6 and Figure 7). Thus, a functional riboflavin biosynthetic capability was observed for all the LAB tested. Moreover, the increase of riboflavin concentration detected in RAMSR supports that there was not a detrimental feedback repression of the vitamin B_2_ synthesis by these LAB when the flavin is present in the environment, as previously observed for *Lactiplantibacillus plantarum* [44]. In addition, for the three *W. cibaria* strains, the absence of riboflavin did not hinder their growth, since almost the same final OD_600nm_ and growth rate were observed in both media (Figure 6). After production, riboflavin secretion took place, since the vitamin was detected in the culture supernatants (Table 2). The production by the three *W. cibaria* strains ranged from 684 to 584 µg/L (Table 2), with the BAL3C-7 strain producing the highest secretion levels. Analysis of the genomic features of *W. cibaria* UC4051 isolated from sorghum flour revealed the presence of the *rib* operon involved in riboflavin synthesis, indicating that this LAB strain could synthesize vitamin B_2_ [67]. To our knowledge, this is the first report of detection of riboflavin production and secretion by a *W. cibaria* strain.

In the case of *L. citreum* BAL3C-4, its growth rate and final biomass were almost identical in RAMS and RAMSR (Figure 7), but its vitamin B_2_ production reached a lower value (325 μg/L) than that of the *W. cibaria* strains (584–684 μg/L) (Table 2). In addition, higher levels of production (1.1 mg/L) have been reported for *L. citreum* CB2567 isolated from kimchi [68]. Finally, the two *L. falkenbergense* strains have similar growth rates in RAMS and RAMSR, but the growth curve of the cultures of the VSL14h-1 strain during the stationary phase in the presence of riboflavin indicated an early lysis (Figure 7). Moreover, during 22 h of growth, VSL14h-1 synthesized 250 μg/L of vitamin B_2_, which was 2.2-fold and approximately 2.5-fold lower levels than VSL11h-8 (548 μg/L) and the *W. cibaria* strains (584–684 μg/L), respectively (Table 2).

Concerning the EPS yields, *W. cibaria* BAL3C-22 produced the highest concentration of EPS (7.4 g/L) after 22 h of incubation, followed by the 2 other *W. cibaria* strains (Table 2). These yields are very high in comparison to dextran production by other *W. cibaria* strains reported by other authors such as 1 g/L EPS [66] or 240 mg/L [58]. *L. citreum* strain produced 4.9 g/L of EPS. These differences in production correlated with the detection of EPS production by TEM (Figure 3 and Figure 4). In addition, very different yields have been reported for the production of dextrans by *L. citreum* strains, ranging from 0.057 g/L [69] to 520 g/L [70]. For *L. falkenbergense* VSL11h-8 and VSL14h-1 strains, the yield was 15- and 30-fold lower than that for *W. cibaria* BAL3C-22, respectively (Table 2). However, to the best of our knowledge, this is the first time that a strain belonging to the *L. falkenbergense* species has been reported to produce an EPS.

### 3.7. Production and Purification of the EPS

The above results revealed that the RAMS medium was suitable not only for riboflavin production but also supported good yields of EPS by the LAB strains. Therefore, in order to characterize the nature of the EPS produced by the six strains, they were grown in the RAMS liquid medium. Then, the EPS were purified from 20 mL culture supernatants after precipitation with one volume of ethanol followed by dialysis steps. We had previously developed this procedure to recover high molecular weight dextrans and to remove contaminants [43]. The three *W. cibaria* strains and *L. citreum* BAL3C-4 produced, respectively, 182, 190, 200, and 122 mg of EPS, and after purification, a recovery of 87% for the four polymers was obtained, without contamination by RNA and proteins (Table 3). Furthermore, the purified EPS produced by the *Weissella* strains were free of detectable DNA contamination, whereas the BAL3C-5 EPS carried 0.026% contamination by this nucleic acid (Table 3). This degree of purity correlates with our previous results during the purification of dextrans produced by other *Leuconostoc* and *Weissella* strains of different origins [11,66] and using the CDM medium instead of RAMS for bacterial growth. The results revealed that the RAMS medium is suitable for the production of pure EPS from *Weissella* and *Leuconostoc.* The use of dextrans in baked goods up to the level of 5% was approved by the European Commission [71]. Moreover, these have the potential to improve dough rheology and bread texture [72]. Therefore, the high degree of purity obtained for the dextrans of *Weissella* and *Leuconostoc* would be very suitable if some of these EPS were to be added during bread making to improve its rheological properties. Other authors have reported dextrans with more than 95% purity after the precipitation step but with some protein residues [11] or DNA residues [66] remaining in the sample. With regard to the *L. falkenbergense* strains, as expected from the initial experiments for cultures grown in RAMS, the EPS production was low: 17 mg for VSL11h-8 and 7 mg for VSL14h-1, with good levels of recovery (76% and 86%), and very low levels (0.09%) of only DNA contamination. The medium and purification protocols seem to be also suitable for the *L. falkenbergense* production of EPS, but the low production indicates that these LAB strains are probably inadequate for either in situ production of the polymer or as a source of the biomolecules for additive usage.

### 3.8. Characterization of the EPS

**Figure 8:**
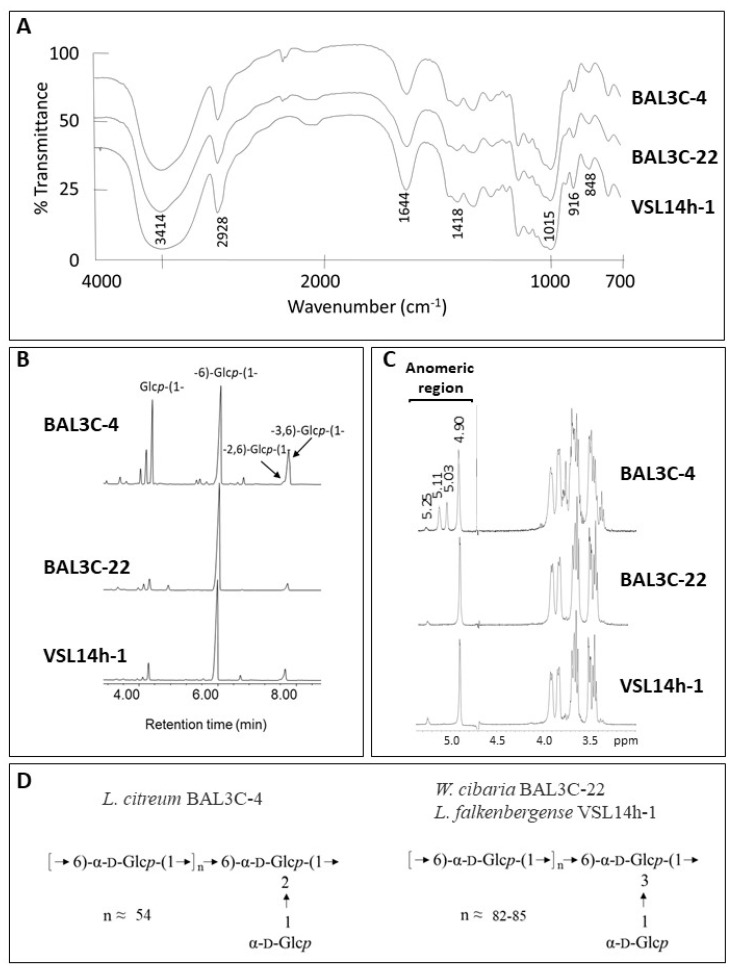
Structural characterization of the dextrans produced by *L. citreum* BAL3C-4, *W. cibaria* BAL3C-22, and *L. falkenbergense* VSL14h-1. (**A**) Infrared spectra of the exopolysaccharides (**B**) EPS bonds deduced from the methylation analysis. (**C**) Representative ^1^H-NMR spectrum of the polysaccharides. (**D**) General structure of the dextrans.

After purification, the EPS from the six LAB strains were subjected to physicochemical characterization, and no intraspecific differences were observed. Therefore, only the results obtained with one strain of each species are depicted in Figure 8. GLC analysis of the monosaccharides released after acid hydrolysis of the EPS indicated that all of them were composed exclusively of glucose. Then, the EPS were analysed by FTIR spectroscopy (Figure 8A), showing the typical profile of polysaccharides [73] and confirming the absence of phosphate, sulphate, or proteins. In addition, the lack of signals characteristic of β-linkages together with the presence of clear absorption bands around 850 cm^−1^ and 920 cm^−1^ indicated that glucose was in the α-configuration [43]. Thus, the EPS are α-glucans. Methylation analysis (Figure 8B) revealed that all of them are indeed dextrans, because they contain a backbone of (1→6)-glucopyranose. However, the degree of branching of the EPS produced by *L. citreum* BAL3C-4 was considerably high. In this polymer, the linear units in the main chain amount to 54%, and the backbone is branched at positions *O*-2 (16%) and *O*-3 (6%) with side chains composed of a single residue of glucopyranose (23%). On the other hand, the branching degree of the dextrans isolated from *W. cibaria* BAL3C-22 and *L. falkenbergense* VSL14h-1 was around 6%.

The results from the previous analyses were confirmed by ^1^H-NMR spectroscopy (Figure 8C). The signals detected in the anomeric region of the spectra were consistent with those reported for dextrans and those observed in Figure S3. The three spectra have in common the presence of a main signal at 4.90 ppm from the linear α-(1→6)-glucopyranose units of the main chain and a second low-intensity peak at 5.3 ppm from the α-(1→3) branches [74,75,76]. In the spectrum from *L. citreum* BAL3C-4, two additional intense signals appear at 5.03 ppm and 5.11 ppm, which have been previously described to correspond to the α-D-Glc*p*-(1→residue from the side chains and the α-2,6-linked-D-glucopyranose branching points in the main chain [40].

The structures deduced for these EPS are depicted in Figure 8D and coincide with those reported by [76] for other *L. citreum* and *W. confusa* isolates, except for the higher degree of branching of the dextrans analysed in this current work.

## 4. Conclusions

In the present work, 22 LAB strains, isolated from different fermented doughs, were identified as belonging to the *Lactobacillus*, *Leuconostoc*, and *Weissella* genera and 21 of them were identified as dextran-producing bacteria. Six strains belonging to three different species *L. citreum*, *W. cibaria*, and *L. falkenbergense* were selected for the screening for EPS and riboflavin production. As far as we know, this is the first instance of *L. falkenbergense* being found in sourdoughs and the first time that dextran production by this species has been investigated. The six LAB strains assayed presented the ability to produce dextran-type EPS and riboflavin. In this context, the *W. cibaria* strains would be the most suitable for dextran and vitamin B_2_ production in situ, and they are also the most suitable to be assayed as potential new starters to obtain functional and bio-fortified breads. Moreover, *L. citreum* BAL3C-4 produces a dextran with an unusually high degree of branching, which could confer specific rheological and/or immunological properties to bread, and it deserves further characterization.

## Figures and Tables

**Table 1:** Summary of the complete species identification of 13 lactic acid bacteria (LAB) representative strains.

Strains Codes	RAPD Type	Closest LAB Type Strains	*rrs* Gene Similarity	*pheS* Gene Similarity
**VSL11h-8**	A	*L. falkenbergense* LMG 10779^T^	100%	99.2%
**VSL14h-1**	B	*L. falkenbergense* LMG 10779^T^	100%	99.2%
mBAL21_1	C	*L. mesenteroides* subsp. *jonggajibkimchii* JCM 6124^T^ (=LMG 6893^T^)	99.9%	100%
**BAL3C-4**	D	*L. citreum* JCM 9698^T^ (=LMG 9849^T^)	99.9%	100%
BAL3C-3	E	*W. cibaria* JCM 12495^T^ (=LMG 17699^T^)	99.9%	98.9%
**BAL3C-5**	F	*W. cibaria* JCM 12495^T^ (=LMG 17699^T^)	99.9%	98.9%
BAL3C-6	G	*W. cibaria* JCM 12495^T^ (=LMG 17699^T^)	99.9%	98.9%
**BAL3C-7**	H	*W. cibaria* JCM 12495^T^ (=LMG 17699^T^)	99.9%	98.9%
BAL3C-9	I	*W. cibaria* JCM 12495^T^ (=LMG 17699^T^)	99.9%	98.9%
BAL3C-11	J	*W. cibaria* JCM 12495^T^ (=LMG 17699^T^)	99.9%	98.9%
BAL3C-12	K	*W. cibaria* JCM 12495^T^ (=LMG 17699^T^)	99.9%	98.9%
BAL3C-13	L	*W. cibaria* JCM 12495^T^ (=LMG 17699^T^)	99.9%	98.9%
**BAL3C-22**	M	*W. cibaria* JCM 12495^T^ (=LMG 17699^T^)	99.9%	98.9%

The specific strains selected for further analysis appear in bold and underlined. ^T^ Type species.

**Table 2:** Exopolysaccharide (EPS) and riboflavin production by lactic acid bacteria grown in RAM medium supplemented with 2% sucrose.

Strain	^1^ EPS (g/L)	^2^ Riboflavin (μg/L)
*W. cibaria* BAL3C-5	6.5 ± 0.9 ^a^	650.3 ± 8.8 ^b^
*W. cibaria* BAL3C-7	6.7 ± 0.8 ^a^	684.5 ± 9.3 ^a^
*W. cibaria* BAL3C-22	7.4 ± 0.9 ^a^	584.3 ± 8.1 ^c^
*L. citreum* BAL3C-4	4.9 ± 0.7 ^b^	325.2 ± 7.3 ^e^
*L. falkenbergense* VSL11h-8	0.58 ± 0.05 ^c^	548.2 ± 9.6 ^d^
*L. falkenbergense* VSL14h-1	0.25 ± 0.04 ^c^	203.5 ± 7.6 ^f^

^1^ EPS concentration was determined from neutral sugars estimation after ethanol precipitation from culture supernatants. ^2^ Riboflavin concentration present in the culture supernatant was inferred from a riboflavin calibration curve. ^a–f^ Means with different letters differed significantly (*p* value ≤ 0.05).

**Table 3:** Purification of EPS produced by LAB.

Strain	Supernatant ^1^					After Precipitation and Dialysis ^2^				
	EPS (mg/mL)	DNA ^3^ (μg/mL)	RNA ^3^ (ng/mL)	Protein (μg/mL)	Total EPS mg	EPS (mg/mL)	DNA ^3^ (ng/mL)	RNA ^3^ (ng/mL)	Protein ^3^ (μg/mL)	Total EPS mg
*W. cibaria* BAL3C-5	7.8 ± 0.01	1.38	<20	110	182	1.0 ± 0.10	60	<20	<1.0	159
*W. cibaria* BAL3C-7	8.2 ± 0.01	0.80	<20	<1.0	190	0.8 ± 0.05	<0.5	<20	<1.0	166
*W. cibaria* BAL3C-22	8.6 ± 0.1	2.51	<20	144	200	0.94 ± 0.12	<0.5	<20	<1.0	175
*L. citreum* BAL3C-4	5.0 ± 0.6	1.07	<20	<1.0	122	0.98 ± 0.05	256	<20	<1.0	107
*L. falkenbergense* VSL11h-8	0.7 ± 0.1	0.63	<20	<1.0	17	0.94 ± 0.13	798	<20	<1.0	13
*L. falkenbergense* VSL14h-1	0.3 ± 0.1	0.30	<20	<1.0	7	0.84 ± 0.16	726	<20	<1.0	6

^1^ Protein, DNA, and RNA concentrations were measured directly from supernatants. Exopolysaccharide (EPS) concentration was determined from neutral sugars estimation after ethanol precipitation from culture supernatants. ^2^ Solutions of EPS were prepared in water at a concentration of 1 mg/mL. ^3^ The contaminants detection limits are 0.5 ng/mL for DNA, 20 ng/mL for RNA, and 1.0 μg/mL for proteins.

## Supplementary Materials

The following are available online at https://www.mdpi.com/article/10.3390/foods10092004/s1, Figure S1. Ropy phenotype of 22 bacterial isolates. Figure S2. RAPD patterns of the 22 LAB strains. Figure S3. 1H-NMR spectra of the EPS produced by representative LAB strains of each RAPD pattern. Figure S4. Detection of evolution of EPS production by LAB in solid medium. Table S1. Results obtained for the 22 bacterial isolates analysed in this study.
