# Supplementary material for: Lactic Acid Bacteria Isolated from Fermented Doughs in Spain Produce Dextrans and Riboflavin
Source: Foods. 2021 Aug 26;10(9):2004. doi: 10.3390/foods10092004 (PMC8470351; doi:10.3390/foods10092004)
Supplement: Supplementary file 1 [file foods-10-02004-s001.zip › foods-1308385-supplementary.pdf]

Table S1. Summary of source, ropy phenotype and phylogenetic typing of the 22 bacterial isolates analysed in this study.

**Isolate#**	**Code**	**Fer-mented dough**	**Ropy phenotype**	**RAPD type**	**Closest LAB species or subspecies**
**VSL11h-8**	1	BD16	+	A	*Leuconostoc falkenbergense*
**VSL14h-1**	2	BD16	+	B	*Leuconostoc falkenbergense*
mBAL21_1	3	MD21	+/-	C	*Leuconostoc mesenteroides* subsp. *jonggajibkimchii*
**BAL3C-4**	4	MD22	++	D	*Leuconostoc citreum*
BAL3C-3	5	MD22	+	E	*Weissella cibaria*
**BAL3C-5**	6	MD22	+	F	*Weissella cibaria*
BAL3C-6	7	MD22	+	G	*Weissella cibaria*
**BAL3C-7**	8	MD22	+	H	*Weissella. cibaria*
BAL3C-9	9	MD22	+	I	*Weissella cibaria*
BAL3C-10	10	MD22	+	F	*Weissella cibaria*
BAL3C-11	11	MD22	+	J	*Weissella cibaria*
BAL3C-12	12	MD22	+	K	*Weissella cibaria*
BAL3C-13	13	MD22	+	L	*Weissella cibaria*
BAL3C-15	14	MD22	++	F	*Weissella cibaria*
BAL3C-16	15	MD22	++	M	*Weissella cibaria*
BAL3C-18	16	MD22	++	M	*Weissella cibaria*
BAL3C-19	17	MD22	++	I	*Weissella cibaria*
BAL3C-20	18	MD22	++	F	*Weissella cibaria*
BAL3C-21	19	MD22	++	G	*Weissella cibaria*
**BAL3C-22**	20	MD22	++	M	*Weissella cibaria*
BAL3C-23	21	MD22	+	G	*Weissella cibaria*
BAL3C-24	22	MD22	+	I	*Weissella cibaria*

The 22 selected lactic-acid bacterial strains and their code numbers in the "PANBAL" (IBFG-CSIC and USAL) or particular IBFG-CSIC collections (Salamanca, Spain) are indicated. The specific strains selected for further analysis are underlined. The bakery-dough (BD) or spontaneously fermented Mother Doughs (MD) of isolation, the respective flours, the bakery or Baker name and the geographical location were as follows: BD16, wheat, Pedro González (Val de San Lorenzo, León, Spain); MD21, wheat, Fred Bakeries (Boulogne-sur-Mer, France); MD22, rye, La Tahona Delicatessen (Salamanca, Spain). ++, strong ropy phenotype; +, normal ropy phenotype; +/- light ropy phenotype. The mucous appearance of the 22 bacterial isolates is shown in Figure S1.

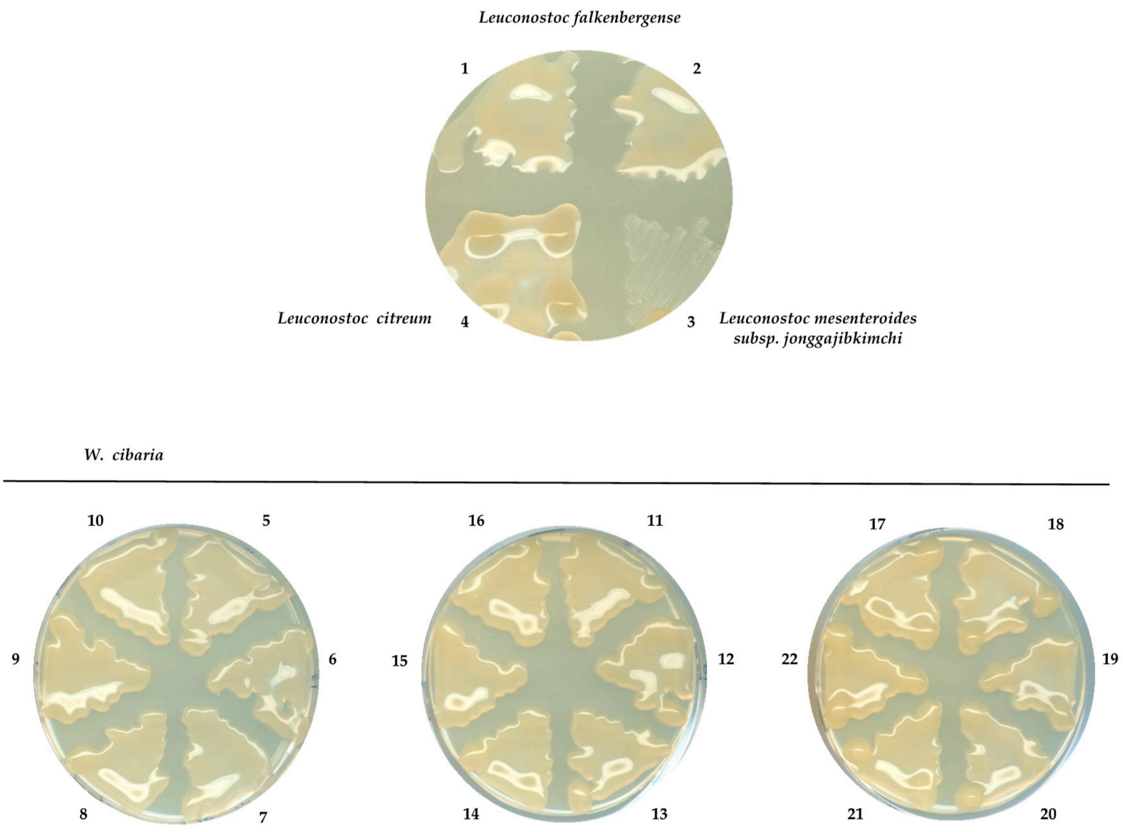

Figure S1. ***Ropy*** **phenotype of the 22 LAB grown on MRS (5% sucrose) plates at 28 °C for 48 h.** The isolate number and the corresponding species are indicated.

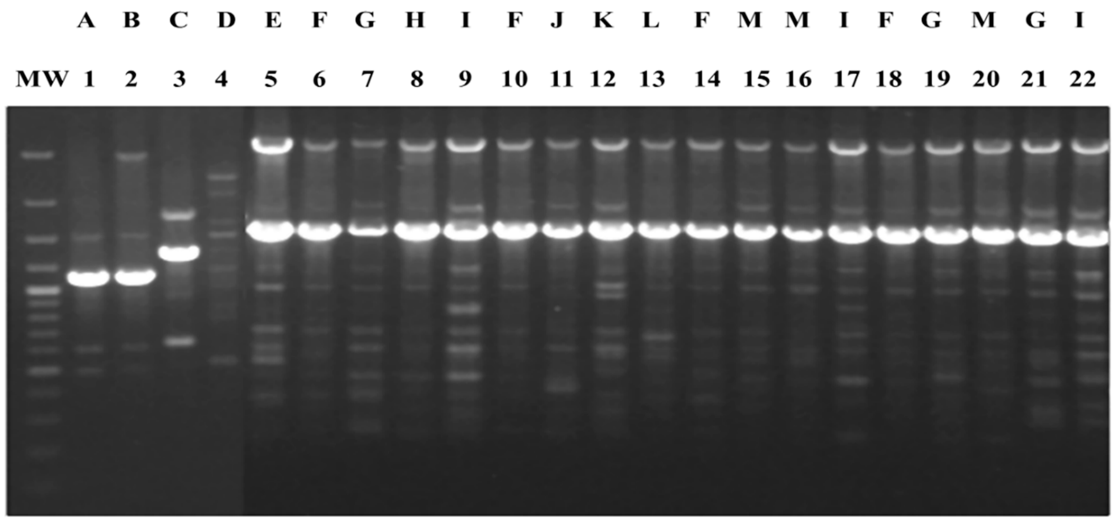

Figure S2. RAPD patterns of the 22 LAB strains. VSL11h-8 (lane 1), VSL14h-1 (lane 2), mBAL21_1 (lane 3), BAL3C-4 (lane 4), BAL3C-3 (lane 5), BAL3C-5 (lane 6), BAL3C-6 (lane 7), BAL3C-7 (lane 8), BAL3C-9 (lane 9), BAL3C-10 (lane 10), BAL3C-11 (lane 11), BAL3C-12 (lane 12), BAL3C-13 (lane 13), BAL3C-15 (lane 14), BAL3C-16 (lane 15), BAL3C-18 (lane 16), BAL3C-19 (lane 17), BAL3C-20 (lane 18), BAL3C-21 (lane 19), BAL3C-22 (lane 20), BAL3C-23 (lane 21), BAL3C-24 (lane 22).

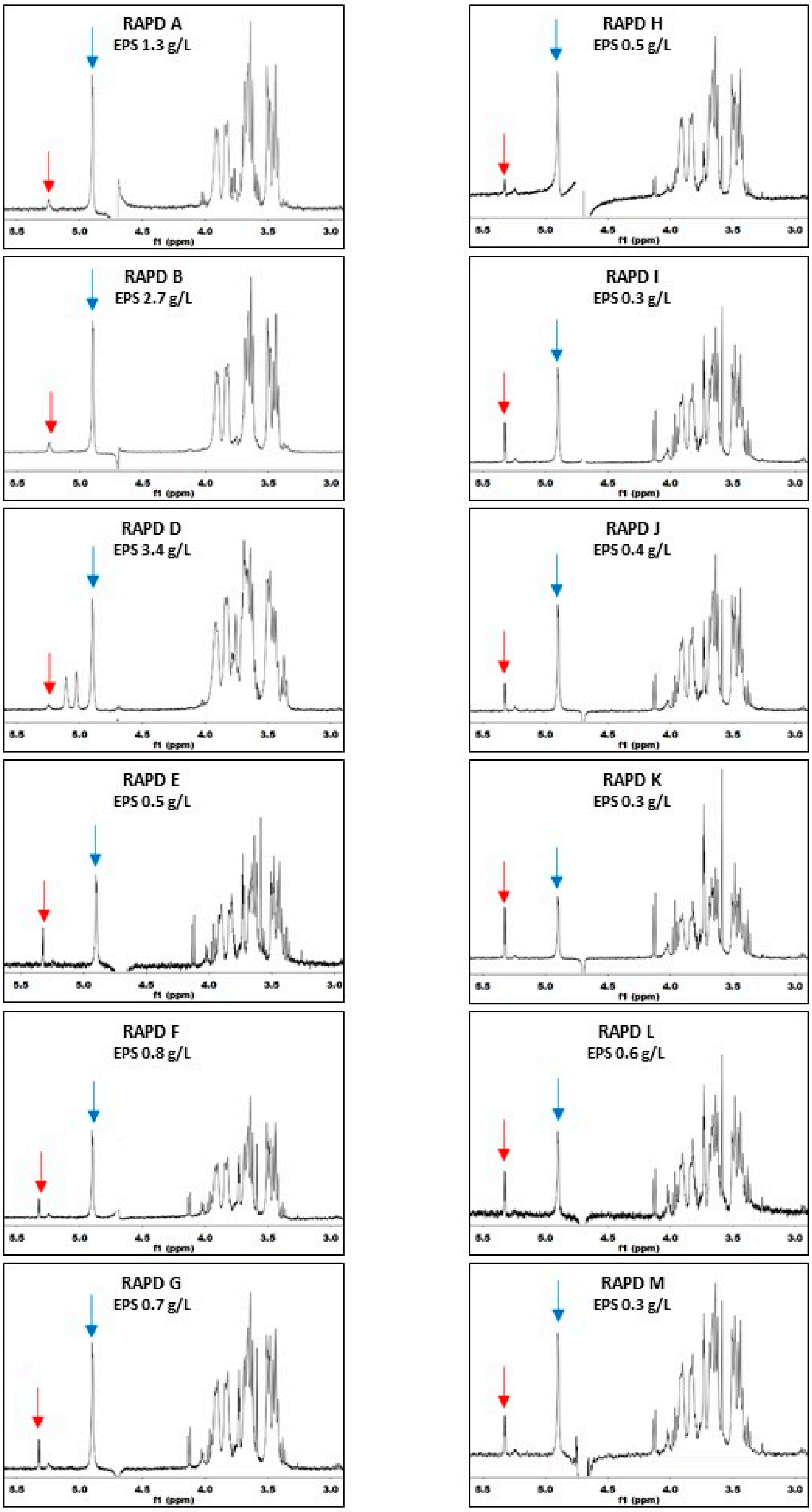

Figure S3. **^{1}H-NMR of EPS produced by LAB grown in SDM medium.** The spectra of polymers synthesized by representative LAB strains of each RAPD pattern are depicted as follows: VSL11h-8 (RAPD A), VSL14h-1 (RAPD B), BAL3C-4 (RAPD D), BAL3C-10 (RAPD E), BAL3C-5 (RAPD F), BAL3C-21 (RAPD G), BAL3C-7 (RAPD H), BAL3C-9 (RAPD I), BAL3C-11 (RAPD J), BAL3C-12 (RAPD K), BAL3C-13 (RAPD L), BAL3C-22 (RAPD M). Arrows in the anomeric region indicate the peak at 4.9 ppm (blue) and at 5.3 ppm (red). The concentrations of EPS after 48 h of bacterial growth are also depicted.

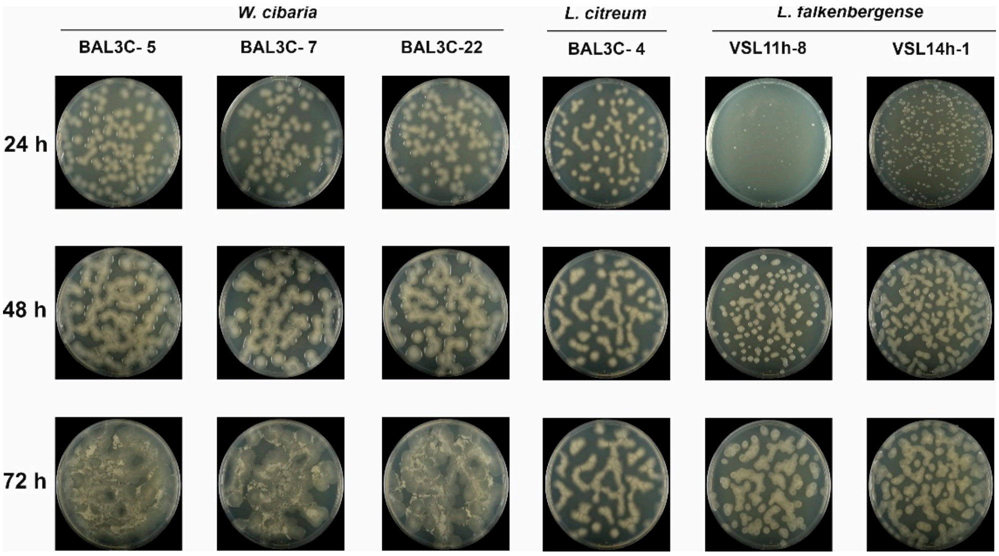

Figure S4. Detection of evolution of EPS production by LAB in solid medium. Pictures of the plates containing LAB grown in MRSS after 24 h, 48 h and 72 h of incubation at 30 °C are depicted.
